# Proteome Dynamics during Antibiotic Persistence and Resuscitation

**DOI:** 10.1128/mSystems.00549-21

**Published:** 2021-08-24

**Authors:** Maja Semanjski, Fabio Lino Gratani, Till Englert, Payal Nashier, Viktor Beke, Nicolas Nalpas, Elsa Germain, Shilpa George, Christiane Wolz, Kenn Gerdes, Boris Macek

**Affiliations:** a Quantitative Proteomics, Interfaculty Institute of Cell Biology, Faculty of Science, University of Tuebingen, Tuebingen, Germany; b Centre for Bacterial Stress Response and Persistence, Section for Functional Genomics, Department of Biology, University of Copenhagen, Copenhagen, Denmark; c Laboratoire de Chimie Bacterienne, CNRS, IMM, Marseille, France; d Medical Microbiology and Hygiene, Faculty of Science, University of Tuebingen, Tuebingen, Germany; Princeton University

**Keywords:** SILAC, bacteria, persistence, proteomics, resuscitation

## Abstract

During antibiotic persistence, bacterial cells become transiently tolerant to antibiotics by restraining their growth and metabolic activity. Detailed molecular characterization of antibiotic persistence is hindered by low count of persisting cells and the need for their isolation. Here, we used sustained addition of stable isotope-labeled lysine to selectively label the proteome during *hipA*-induced persistence and *hipB*-induced resuscitation of Escherichia coli cells in minimal medium after antibiotic treatment. Time-resolved, 24-h measurement of label incorporation allowed detection of over 500 newly synthesized proteins in viable cells, demonstrating low but widespread protein synthesis during persistence. Many essential proteins were newly synthesized, and several ribosome-associated proteins such as RaiA and Sra showed high synthesis levels, pointing to their roles in maintenance of persistence. At the onset of resuscitation, cells synthesized the ribosome-splitting GTPase HflX and various ABC transporters, restored translation machinery, and resumed metabolism by inducing glycolysis and biosynthesis of amino acids.

**IMPORTANCE** While bactericidal antibiotics typically require actively growing cells to exploit their function, persister cells are slowly replicating which makes them tolerant to the lethal action of antimicrobials. Here, we used an established *in vitro* model of bacterial persistence based on overexpression of the paradigm toxin-antitoxin (TA) system *hipA*/*hipB* to devise a generic method for temporal analysis of protein synthesis during toxin-induced persistence and antitoxin-mediated resuscitation. Our time-resolved, 24-h measurement of label incorporation demonstrated low but widespread protein synthesis during persistence. At the onset of resuscitation, cells restored translation machinery and resumed metabolism by inducing glycolysis and biosynthesis of amino acids. Our study provides the first global analysis of protein synthesis in persisting and resuscitating bacterial cells, and as such, presents an unprecedented resource to study the processes governing antibiotic persistence.

## INTRODUCTION

Antibiotic resistance is an acute health problem. Many bacteria are categorized as serious threats representing a considerable clinical and financial burden (1, 2). In addition to resistance, bacteria also possess an elusive innate strategy, termed persistence, that enables them to tolerate antibiotics without acquiring genetic changes (3). Persisters are defined as phenotypic variants of bacterial cells that become transiently tolerant to antibiotics by restraining their growth and entering a dormant-like state (4, 5). Persistence is exhibited within a subpopulation of genetically uniform cells, a phenomenon present in all bacteria tested so far (6, 7). While bactericidal antibiotics typically require actively growing cells to exploit their function, persister cells are slowly replicating which makes them tolerant to the lethal action of antimicrobials. In addition to slow growth, the persister phenotype is also associated with very low metabolic activity (8, 9).

Persistence can be induced by toxin-antitoxin (TA) modules that usually consist of two genes: one encoding a toxin that inhibits cell growth and another encoding an antitoxin that inhibits toxin activity (10, 11). While the product of the toxin gene is a protein that interferes with essential cellular functions, antitoxin genes encode either small proteins or noncoding RNAs (10). Several studies have shown that persister cultures are heterogenous, with large abundance of cells with intact membranes that are viable but nonculturable (VBNC) (12–15). In addition, low counts of persister cells in these cultures necessitate application of elaborate protocols for their isolation (16–18), making detailed, unbiased exploration of the unperturbed molecular processes a challenging task.

Here, we used an *in vitro* model of bacterial persistence based on conditional overproduction of HipA toxin and HipB antitoxin (19–24) to devise a generic method for temporal analysis of protein synthesis during toxin-induced persistence and antitoxin-mediated resuscitation. The bacterial serine-threonine protein kinase HipA promotes multidrug tolerance by phosphorylating the glutamate-tRNA ligase (GltX), leading to a halt in translation, inhibition of growth, and induction of a physiologically dormant state (persistence) (19–21). We reasoned that prolonged antibiotic treatment of HipA-induced Escherichia coli cells, which results in a mixture of dead (lysed) and viable cells, can be coupled with the sustained addition of stable isotope-labeled amino acids directly to the culture. As only the viable, persisting cells are capable of incorporating the label and the label mass shift can be easily resolved in a mass spectrometer, this approach can be used to selectively label and detect newly synthesized proteins in the high background of proteins originating from dead cells. For simplicity, in this paper, we will use the term “persisters” for all cells that remain viable after this treatment; however, we note that this population is heterogenous and consists of culturable persisters and viable but nonculturable cells. Similar strategies, termed “pulsed” or “dynamic” stable isotope labeling by amino acids in cell culture (SILAC), have previously been used to analyze protein turnover in eukaryotic (25, 26) and prokaryotic cells (27, 28).

## RESULTS

### HipA-induced persister cells show low but measurable protein synthesis.

**FIG 1:**
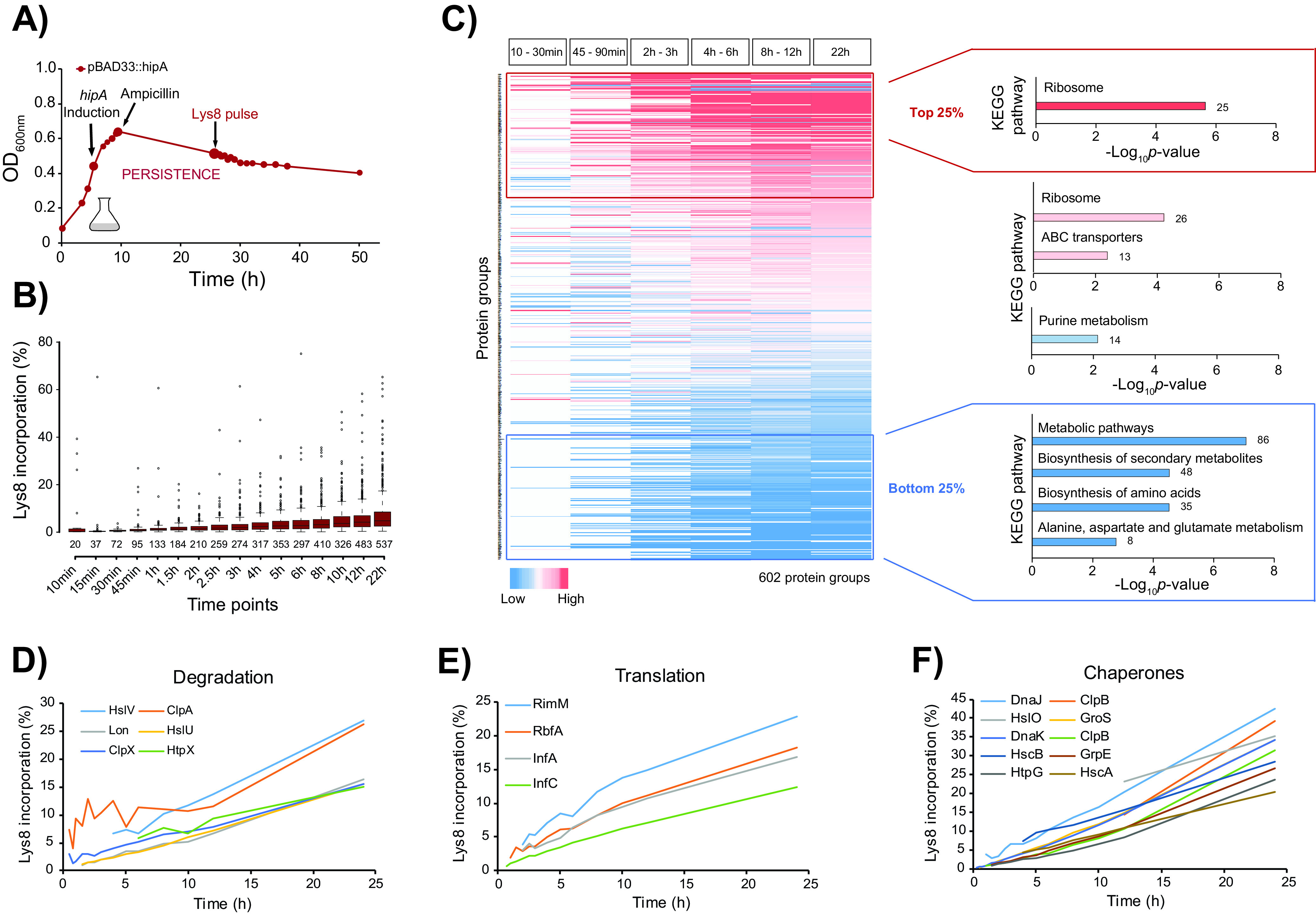
Persister cells synthesize proteins involved in essential physiological processes. (A) Transcription of plasmid-encoded *hipA* (pBAD33::*hipA*) was induced in E. coli K-12 strain MG1655 at an OD_600_ of 0.4 for 3 h, followed by ampicillin treatment and addition of glucose to repress further *hipA* transcription. Approximately 20 h after *hipA* induction, the culture was sampled at 16 time points. Proteins were digested with endoproteinase Lys-C, and peptides were identified and quantified by LC-MS/MS. The growth curve is representative of three biological replicates. (B) Incorporation of Lys8 into proteins across 17 time points calculated from the measured protein H/L ratios. The box plots are representative of three biological replicates. (C) Heat map of all quantified proteins color coded based on their H/L ratio ranking within each time bin. Missing values are colored in white. KEGG pathway enrichment analysis of quanified proteins in the 25th percentile (top 25% [red]), 25th to 50th percentile (pink), 50th to 75th percentile (light red and blue), and 75th percentile (bottom 25% [blue]) across all time bins. The number of enriched proteins in each category is indicated outside the bars. (D to F) Label incorporation curves of selected examples of enzymes involved in protein degradation, translation, and folding (chaperones).

To test applicability of our approach, we first increased the number of persister cells growing in minimal medium by ectopically inducing *hipA* in E. coli MG1655. Three hours after HipA-induced growth inhibition, we treated the culture with ampicillin to lyse antibiotic-sensitive cells. During ampicillin treatment, we repressed expression of *hipA* by the addition of glucose to prevent constant HipA overproduction. Twenty hours after ampicillin treatment, we added ^15^N_2_^13^C_6_-lysine (Lys8) to the culture; this time point was sufficient to eradicate sensitive cells and achieve a steady state of persistence, as demonstrated by approximately 1,000-fold higher count of CFU in the *hipA*-induced strain compared to the empty vector (see Fig. S1A and B in the supplemental material). After introduction of the Lys8 label, we harvested the culture aliquots by brief centrifugation to pellet intact cells in three biological replicates at multiple time points ranging from 10 min to 24 h (Fig. 1A). In this experimental setup, only viable cells incorporated Lys8, which enabled specific detection of newly synthesized proteins, temporal quantification of protein abundance, and estimation of protein half-lives (see Fig. S1C and Data Set S1, sheet 1, in the supplemental material). The number of identified proteins across time points was consistent, with 1,993 proteins identified on average at each time point (Fig. S1D). Remarkably, up to 531 proteins have partially incorporated the Lys8 label and were reproducibly quantifiable in the high background of Lys0-labeled proteins originating from dead cells (Fig. S1D and S1E). Due to HipA-mediated inhibition of cell growth, incorporation of Lys8 in these proteins was only partial and expected to be very low; 24 h after the start of labeling, the average intensity of all heavy labeled signals amounted to only 8.64% of the total ion intensity (Fig. 1B). Nevertheless, the measurable label incorporation was indicative of protein synthesis in viable cells and was in agreement with previous studies (28–30). The low level of label incorporation reflected the low turnover rates and long half-lives of proteins in persisting cells. Estimation of protein half-lives from the measured heavy/light (H/L) ratios as described previously (25) revealed that the median protein half-life under these conditions was longer than 250 h (Fig. S1G and Data Set S1, sheet 1). This, however, is only an estimate, since our measurement window (24 h) was significantly shorter than the actual protein half-life, and our calculation was based on the assumption of complete cell growth arrest. This experiment demonstrated that it is possible to partially label and directly detect newly synthesized proteins of viable cells after toxin overproduction and antibiotic treatment. All measured proteins and their ratios are listed in Data Set S1, sheet 2, and can be browsed online at https://pctsee.pct.uni-tuebingen.de.

**FIG 2:**
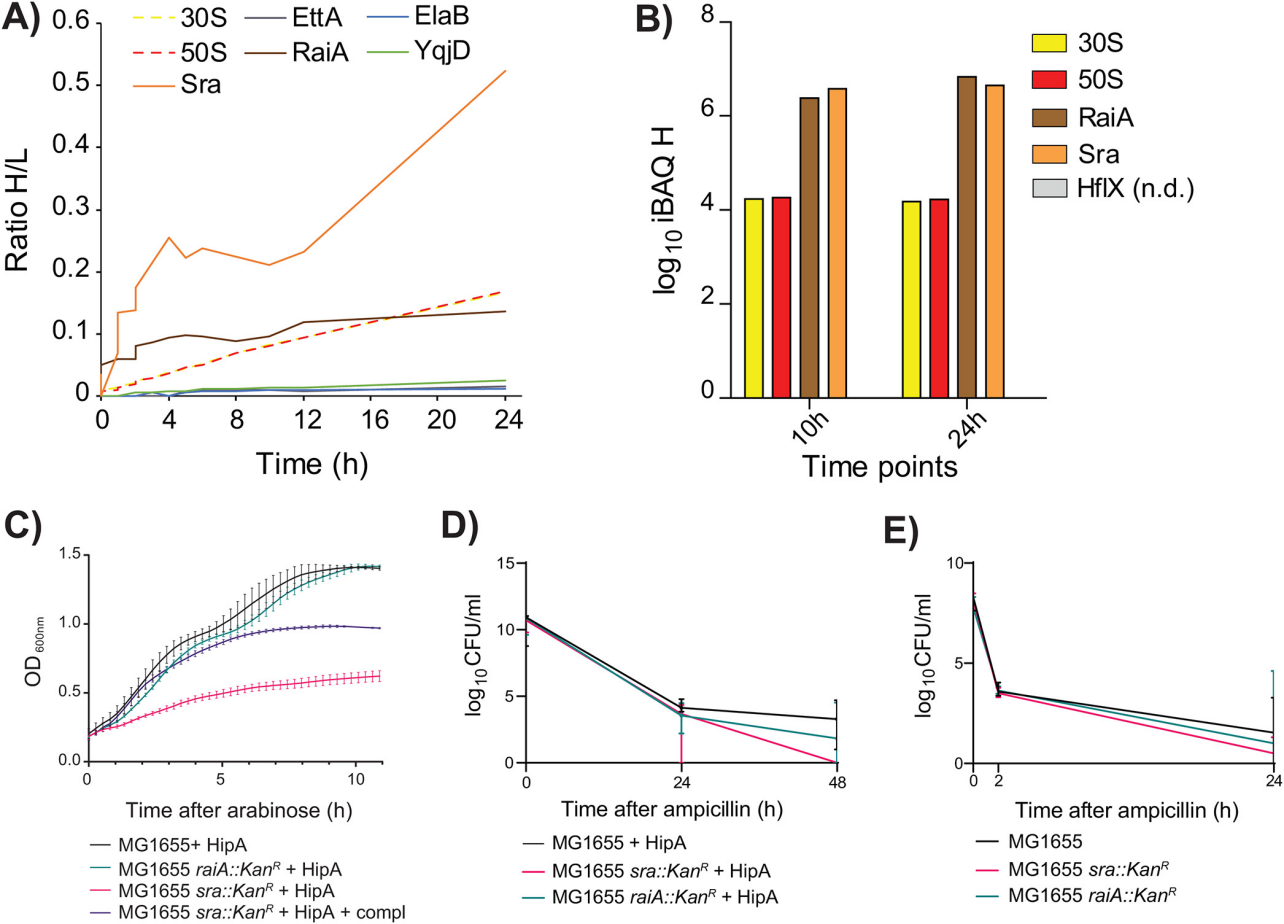
Ribosome-associated proteins RaiA and Sra show elevated synthesis levels in persisting cells and a role during antibiotic tolerance. (A) All detected ribosomal proteins had comparable label incorporation rates. The synthesis rate of the hibernation factor RaiA was elevated and similar to ribosomal proteins, whereas the synthesis rate of the ribosome-associated protein Sra was markedly higher than that of the ribosomal proteins. Most of the ribosome-associated hibernation factors (e.g., EttA, ElaB, YqjD) showed very low synthesis levels. (B) Estimates of the amounts of newly synthesized proteins based on intensity-based absolute quantification (iBAQ) (25) in the heavy SILAC channel. The amount of newly synthesized RaiA and Sra proteins was about 100 times higher than all ribosomal proteins combined. Depicted are the median values of three biological replicates. n.d., not determined. (C) Wild-type (WT) and Δ*raiA* deletion strain presented similar growth phenotypes upon HipA ectopic expression, while strain Δ*sra* deletion showed a stronger growth inhibition upon HipA expression, which can be partially complemented by plasmid bearing *sra* gene with its native promoter. Strains bearing with empty vectors are used as controls. (D) WT strains ectopically expressing HipA were more viable compared to the same strain bearing the empty vector. Two deletion strains (Δ*sra* and Δ*raiA*) expressing *hipA* showed a strong decrease in CFU, indicating a potential role of Sra and RaiA in the antibiotic tolerance mechanism. Control experiments including expression of empty vector are presented in Fig. S1H in the supplemental material. Equivalent experiments in the M9 medium are shown in Fig. S1I. (E) Without HipA ectopic overexpression, only the mutant lacking the *sra* gene showed a lower survival in the LB medium.

10.1128/mSystems.00549-21.1 FIG S1 Measurement of newly synthesized proteins in persisting E. coli cells. (A) OD_600_ measurements of the E. coli cultures after induction of ectopic *hipA* expression compared to the empty vector. (B) Colony-forming unit (CFU) measurements of the *hipA*-expressing E. coli culture at several time points before and after ampicillin addition. E. coli cells containing empty vector were used as a control. (C) Biochemical and mass spectrometry workflow employed in our study. (D) Lys8 label incorporation monitored by increasing number of quantified proteins over 24 h. Quantified proteins were detected in both “light” (“L”) and “heavy” (“H”) form, which enabled calculation of their H/L ratios (relative quantification). (E) Correlation plot demonstrates high reproducibility between two biological replicates. (F) Estimated half-lives of newly synthesized proteins during persistence. (G) Empty vector controls for HipA growth effect measurements in LB medium (Fig. 2C). (H) Empty vector controls for persistence measurements in LB medium (Fig. 2D). (I) CFU measurement of the wild type and strains lacking *sra* and *raiA* before and after ampicillin addition, in M9 medium. Download FIG S1, TIF file, 1.1 MB. Copyright © 2021 Semanjski et al. 2021 Semanjski et al. https://creativecommons.org/licenses/by/4.0/ This content is distributed under the terms of the Creative Commons Attribution 4.0 International license.

10.1128/mSystems.00549-21.5 DATA SET S1 (Sheets 1 to 5) HipA-induced persistence: protein half-lives (sheet 1), protein groups (sheet 2), GO term enrichment (sheet 3), protein groups (union of replicates) (sheet 4), and ribosomal proteins (sheet 5). (Sheets 6 to 10) HipB-induced resuscitation: protein groups (sheet 6), protein groups (union of replicates) (sheet 7), GO term enrichment (sheet 8), cluster overrepresentation (sheet 9), and ribosomal proteins (sheet 10). Download Data Set S1, XLSX file, 10.4 MB. Copyright © 2021 Semanjski et al. 2021 Semanjski et al. https://creativecommons.org/licenses/by/4.0/ This content is distributed under the terms of the Creative Commons Attribution 4.0 International license.

### Persisting bacteria produce proteins needed for essential cellular processes.

For further data analysis, the time points over the entire 24-h course of persistence were collapsed into six time bins, and proteins were ranked based on their H/L ratios. Gene Ontology (GO) analysis of all newly synthesized proteins showed that proteins involved in translation, glycolysis, protein targeting, redox homeostasis, and tricarboxylic acid cycle were significantly enriched (*P* < 0.01) (Data Set S1, sheet 3). Importantly, almost all ribosomal proteins were overrepresented among the top 25% proteins that exhibited relatively high label incorporation across all time points (*P* = 1.67E−15). In contrast, proteins with lower label incorporation (bottom 25%) participate mainly in the metabolic pathways such as biosynthesis of amino acids and other metabolites (Fig. 1C). Interestingly, enzymes involved in protein degradation, including degradation of antitoxins necessary to maintain an optimal toxin/antitoxin ratio during persistence, also exhibited higher label incorporation. They included several proteases (Lon, HslU, HslV, and HtpX), as well as the specificity components of the Clp protease (ClpA and ClpX, but not ClpP), implying that protein degradation is active during persistence (Fig. 1D). Two antitoxins, PrlF and MqsA, as well as several heat and cold shock proteins involved in stress response, also showed Lys8 incorporation. Furthermore, proteins involved in transcription, such as the ribonucleases RNase R (Rnr) and RNase III (Rnc) required for RNA processing and turnover, including the RNA polymerase sigma factor RpoD, were also detected as newly synthesized, as were several translation regulatory proteins essential for the start of protein synthesis, such as translation initiation factors IF-1 and IF-3 (InfA and InfC) (Fig. 1E). Several chaperones were detected as newly synthesized during persistence (Fig. 1F), suggesting that they may be required for the maintenance of proteome homeostasis, either by enabling correct folding of misfolded client proteins or by targeting unfolded (or aggregated) proteins for degradation. Among them were DnaK, DnaJ, and ClpB, that were previously connected to persistence (31, 32). Moreover, one of the most important DNA repair proteins RecA was identified as newly synthesized in our study, which is in agreement with a previous report that SOS response genes are expressed during persistence due to inhibited DNA replication (32). These results indicate that bacterial cells perform active translation during antibiotic persistence and produce proteins that are needed for essential cellular processes. In fact, out of 299 essential gene products in E. coli, 159 incorporated the label during persistence (Data Set S1, sheet 4).

### Ribosome-associated proteins RaiA and Sra show elevated synthesis levels during persistence.

Consistent with the slow growth attributed to persister cells, proteins required for cell division (FtsH, FtsZ, FtsY, and ZipA) were either not detected or displayed very low label incorporation in our data set, suggesting that residual cell growth is still present but is extremely slow. Accordingly, chemotaxis-related proteins that are important for cell motility were not identified, which supports the absence of cell movement during persistence. The low abundance of newly synthesized proteins involved in metabolism, mainly in biosynthesis of amino acids and primary carbon metabolism, demonstrated that major energy-generating pathways are active in our model of persistence, but their activity is very low. In contrast to reduced metabolism and cell division, we found that ribosomal proteins and general stress response-related proteins were incorporating the Lys8 label to a higher extent during persistence.

Regulation of ribosome function is known to have a key role in bacterial persistence (33); therefore, we next analyzed relative abundance and label incorporation of all detected ribosomal proteins, as well as ribosome-associated hibernation and stationary-phase factors proposed to modulate ribosomal activity, such as EttA, RaiA, ElaB, YqjD, and Sra (33–35). In persisting cells, measured rates of label incorporation and relative abundances of most ribosomal proteins were relatively high (Fig. 2A). Of hibernation factors, only RaiA was detected as newly synthesized at a relatively high rate, pointing to its potential role as a major factor in active maintenance of ribosome hibernation during persistence. Intriguingly, the stationary-phase ribosome-associated factor Sra showed an even higher label incorporation rate (Fig. 2A). Importantly, the level of newly synthesized GTPase HflX, proposed to mediate activation of hibernating ribosome dimers (36), was not detectable during persistence. Strikingly, the overall abundance of newly synthesized hibernation factors (predominantly RaiA), estimated using intensity-based absolute quantification (iBAQ) of the heavy isotope channel, was about 100-fold higher than the level of all newly synthesized ribosomal proteins combined (Fig. 2B and Data Set S1, sheet 5).

### Ribosome-associated protein Sra is involved in antibiotic tolerance.

To analyze potential involvement of Sra and RaiA in the HipA-induced growth inhibition, we induced *hipA* in the *sra* and *raiA* deletion mutants at the beginning of the exponential phase and followed their growth over time (Fig. 2C and Fig. S1G). While the *raiA* mutant showed a growth curve comparable to that of the wild type (WT) upon *hipA* induction (Fig. 2C), the *sra* mutant showed a stronger inhibition, which could be partially complemented with a plasmid bearing *sra* with its native promoter (Fig. 2C). To further test whether ribosome-associated proteins Sra and RaiA influence HipA-induced persistence and antibiotic tolerance, we ectopically induced *hipA* in E. coli *sra* and/or *raiA* deletion strains in log phase and treated the cultures grown in LB medium with ampicillin for up to 48 h. Already 24 h after the addition of antibiotic, the CFU counts of the HipA-producing *sra* and *raiA* mutants were lower than the *hipA*-expressing WT, pointing to a lower tolerance of these strains to ampicillin (Fig. 2D and Fig. S1H). A similar effect was also observed in M9 minimal medium (Fig. S1I). The difference in the CFU count was even more pronounced 48 h after the addition of antibiotic, with the CFU count of the *raiA* mutant about 100-fold lower and that of the *sra* mutant 1,000-fold lower than that of the WT. A less pronounced but similar trend was also observed when *hipA* was not induced (Fig. 2E). These experiments strongly suggested involvement of ribosome-associated proteins, especially Sra, in the maintenance of antibiotic tolerance.

### HipB production leads to rapid resuscitation and incorporation of the Lys8 label.

**FIG 3:**
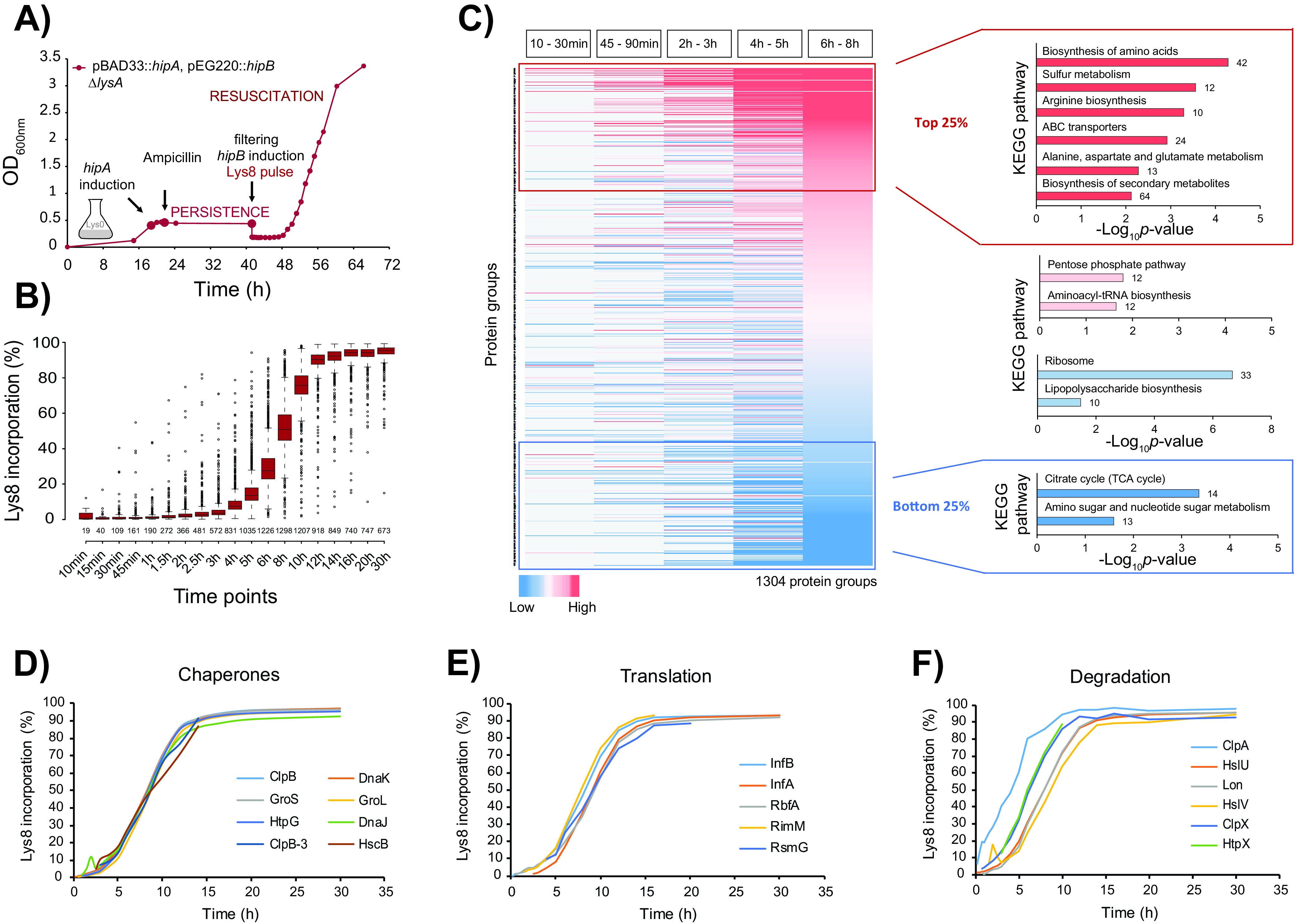
Resuscitating cells restore metabolic activity and translation machinery. (A) Transcription of *hipA* was induced and repressed as described in the legend to Fig. 1. To trigger resuscitation, a culture sample was filtered, and cells were transferred into fresh medium containing IPTG to induce expression of *hipB* (*p_lac_*::*hipB*) and “heavy” lysine (Lys8) for pulse-labeling. Cells were harvested at 21 time points thereafter. The growth curve is representative of three biological replicates. (B) Incorporation of Lys8 into proteins across 19 time points calculated from the measured protein H/L ratios. The box plots are representative of three biological replicates. (C) Heat map of all quantified proteins color coded based on their H/L ratio ranking within each time bin. Missing values are colored in gray. KEGG enrichment analysis of proteins in the 25th percentile (top 25% [red]), 25th to 50th percentile (pink), 50th to 75th percentile (light red and light blue), and 75th percentile (bottom 25% [blue]) across all time bins was performed with DAVID software against the background of all quantified proteins. The number of enriched proteins in each category is indicated outside the bars. TCA, tricarboxylic acid. (D to F) Label incorporation curves of selected examples of enzymes involved in protein and folding (chaperones), translation, and degradation.

We next applied our approach to study proteins produced upon induction of resuscitation. As in the first experiment, we increased the number of persister cells by ectopically inducing *hipA* in minimal medium supplemented with Lys0 and treated the culture with a high dose of ampicillin. We repressed expression of *hipA* during ampicillin treatment by adding glucose to ensure steady-state conditions independent of any HipA overproduction. To trigger resuscitation, we removed ampicillin, Lys0, and cellular debris of lysed cells by gentle filtration and added fresh minimal medium containing β-d-1-thiogalactopyranoside (IPTG) to induce transcription of *hipB* to produce HipB antitoxin. The medium also contained “heavy” lysine (Lys8) for metabolic labeling of resuscitating cells. Filtered cultures exposed to the fresh, IPTG-containing medium resumed growth, whereas unfiltered control cultures did not (Fig. S2A), demonstrating that resuscitation was driven by *hipB* induction. The resuscitating culture was harvested in three biological replicates and 20 time points (Fig. 3A). Incorporation of Lys8 in resuscitating cells followed the shape of the growth curve and saturated at average 94.6% (Fig. 3B). By 30 min after induction of resuscitation, more than 100 proteins incorporated the Lys8 label and could be quantified. Although the median of Lys8 incorporation during the first 4 h was low (<8%), the presence of outliers indicated that a subset of proteins such as NemA, FrmB, and BhsA incorporated the label faster than others and are therefore likely to play a role in resuscitation (Data Set S1, sheet 6). On average, 1,880 proteins were identified at an estimated false discovery rate (FDR) of 1.3% (Fig. S2B). The three biological replicates showed good reproducibility of measured protein SILAC ratios across all time points (Fig. S2C).

10.1128/mSystems.00549-21.2 FIG S2 Measurement of newly synthesized proteins in resuscitating E. coli cells. (A) OD_600_ measurements of *hipB*-induced and -uninduced (“no filter”) cultures confirm that resuscitation was driven by ectopic *hipB* expression. In the absence of *hipB* expression, the OD_600_ remained constant for at least 30 h, demonstrating that no spontaneous resuscitation or development of resistance occurred during this time period. (B) Lys8 label incorporation monitored by increasing number of quantified proteins over 30 h. Note that after 8 h, most proteins are predominantly present in their heavy form and their H/L ratio cannot be calculated, which leads to an apparent “decrease” in the number of quantified proteins. (C) Correlation plot of two biological replicates demonstrates high reproducibility. (D) Abundance traces of ribosomal proteins L31 (*rpmE*) and L36 (*rpmJ*), and their paralogs L31-type B (*ykgM*) and L36-2 (*ykgO*) point to a rearrangement of the large ribosomal unit at the entrance to stationary phase. Incomplete profiles are caused by missing values in the data set. Download FIG S2, TIF file, 0.9 MB. Copyright © 2021 Semanjski et al. 2021 Semanjski et al. https://creativecommons.org/licenses/by/4.0/ This content is distributed under the terms of the Creative Commons Attribution 4.0 International license.

To analyze processes occurring early during resuscitation, we considered time points from the first 8 h of *hipB* induction, in which the optical density doubled. Measured protein ratios were collapsed into five time bins, and proteins were ranked based on their H/L ratio within each time bin. According to the KEGG pathway enrichment analysis, proteins exhibiting high label incorporation (top 25%) during the entire 8-h course of resuscitation are involved mainly in the biosynthesis and transport of amino acids, such as arginine and cysteine, as well as in alanine, aspartate, and glutamate metabolism (*P* value of <0.05). Conversely, proteins with a low label incorporation (bottom 25%) are constituents of the citric acid cycle and amino sugar and nucleotide sugar metabolism (Fig. 3C). Altogether, this implies that resuscitating bacteria are predominantly synthesizing enyzmes for anabolic pathways to produce building blocks for cell growth. Proteins involved in catabolic pathways that consume metabolites to release energy are synthesized at a lower rate compared to all quantified proteins. This is in agreement with our experimental design, as we were resuscitating cells with glucose as the carbon source. Therefore, most energy was derived from glucose catabolism (glycolysis), not from the catabolism of other metabolites.

### The onset of resuscitation is marked by restoration of glycolysis and the translation machinery.

To identify proteins that are potentially regulating the switch from persistence to resuscitation, the first time bin (10 to 30 min) after induction of *hipB* was analyzed in more detail. GO enrichment analysis revealed that 43 out of 153 quantified proteins take part in translation (*P* = 2.75E−27), out of which 42 are ribosomal proteins and one is Sra (Data Set S1, sheets 7 and 8). Glycolysis pathway, stress response, and protein folding were also significantly enriched. This implies that, at the very onset of resuscitation, cells restored almost the entire translation machinery and restarted their metabolism by inducing glycolysis to convert glucose from the fresh medium into energy used for regrowth (Data Set S1, sheet 8). Several proteins showed unusually high label incorporation in the first 30 min after induction of resuscitation. As expected, among them was HipB antitoxin that was produced from the plasmid and therefore served as a positive control. Other proteins participate in various processes, such as the ribonucleotide monophosphatase NagD that dephosphorylates a wide range of (deoxy)ribonucleoside phosphates, or the *S*-formylglutathione hydrolase FrmB, which converts *S*-formylglutathione into formate and glutathione to detoxify formaldehyde that can otherwise chemically modify DNA and proteins (37). Among them were also several proteins known to play a role in persistence, such as the ATP-dependent Clp protease subunit ClpA that directs the ClpAP protease to specific substrates for their degradation (38, 39) (Fig. 3F) and the RNA polymerase sigma factors RpoS and RpoD. RpoS is important for transcriptional reprogramming of many genes that are mainly involved in the metabolism and stress response (39), whereas RpoD preferentially induces transcription of genes associated with fast growth, such as ribosomal proteins (40), which is in agreement with our findings (see below). Considering that RpoD was also detected during persistence, it can be assumed that it might have a dual role during both processes. Moreover, several other proteins were rapidly synthesized during early resuscitation, such as NemA, which reduces *N*-ethylmaleimide that inhibits growth by modifying cysteine residues of cellular proteins (41). NemA is also known to degrade toxic compounds to use them as a source of nitrogen (42) (Fig. S4).

10.1128/mSystems.00549-21.4 FIG S4 Map of top 25 quantified proteins with highest incorporation of Lys8 in each time bin during resuscitation. Different functional descriptions are color coded according to the legend. Boxes around individual proteins are coded based on the style of the line and indicate the confidence of protein quantification per time bin according to the following classification system: protein is quantified in >67% of the time points in three replicates (class I), protein is quantified in 33 to 67% of the time points in three replicates (class II), and protein is quantified in <33% of the time points in three replicates (class III). Download FIG S4, TIF file, 0.7 MB. Copyright © 2021 Semanjski et al. 2021 Semanjski et al. https://creativecommons.org/licenses/by/4.0/ This content is distributed under the terms of the Creative Commons Attribution 4.0 International license.

### Resuscitation is a concerted and tightly regulated biological process.

To obtain global insight into processes occurring during resuscitation, we performed statistical enrichment analysis of KEGG- and GO-annotated functions in each of the five time bins. Functions related to ribosome and protein biosynthesis were significantly enriched in all time bins, demonstrating that translation is the key process that dominates resuscitation. In addition, the following protein functions were overrepresented: bin 1 (10 to 30 min), glycolysis and chaperone-mediated protein folding; bin 2 (45 to 90 min), glycolysis and aminoacyl-tRNA synthetase; bin 3 (2 to 3 h), biosynthesis of antibiotics and amino acids; bin 4 (4 to 5 h) and bin 5 (6 to 8 h), biosynthesis of amino acids, antibiotics, and carbon metabolism (Data Set S1, sheet 8). For 1,663 proteins, we were able to measure label incorporation over all 21 measured time points. Unsupervised clustering of their temporal profiles revealed eight distinct clusters that differed markedly in the rate of label incorporation, estimated by the average time needed to incorporate 50% of the label (Fig. S3 and Data Set S1, sheet 9). Among proteins with fast label incorporation, functions such as “ABC transporters” and “arginine biosynthetic process” were overrepresented (“early functions”). They were followed by protein clusters enriched in diverse metabolic pathways and protein biosynthesis (“intermediate functions”). Proteins with the slowest label incorporation were predominantly involved in various catabolic processes; these “late functions” most likely do not play a role in resuscitation but may be of importance in the transition to stationary phase that was also covered in our analysis. Combined, this analysis revealed that resuscitation is a tightly regulated biological process that starts with activation of protein translation and synthesis of transporters needed to import nutrients and building blocks for protein biosynthesis.

10.1128/mSystems.00549-21.3 FIG S3 Clustering of label incorporation profiles allows reconstruction of time-dependent cell metabolism during resuscitation. Heavy label incorporation was used to cluster proteins across the resuscitation time scale. Among the eight clusters represented, clusters 8, 4, and 1 belong to the early synthesized proteins, clusters 5, 3, and 2 belong to the medium proteins, and clusters 6 and 7 belong to the late proteins. The smoothed conditional means are represented as a dashed curve within each cluster. For each cluster, the time at 50% incorporation is determined based on the intersection with smoothed means. The top significantly overrepresented functional category for each cluster (FDR ≤ 0.05) are plotted on the right panels. Download FIG S3, TIF file, 1.9 MB. Copyright © 2021 Semanjski et al. 2021 Semanjski et al. https://creativecommons.org/licenses/by/4.0/ This content is distributed under the terms of the Creative Commons Attribution 4.0 International license.

### Ribosomal hibernation factor HflX is induced in resuscitating cells.

**FIG 4:**
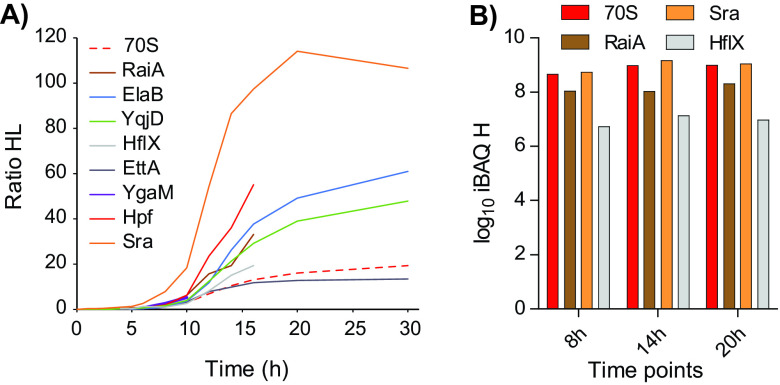
Ribosomal proteins but also hibernation factors are heavily synthesized in resuscitating cells. (A) Label incorporation rates of ribosomal proteins and selected hibernation factors during resuscitation. All ribosomal proteins, except L31-B and L36-2, showed concerted synthesis levels (dashed red trace). The hibernation factor HflX was induced at the onset of resuscitation. Remarkably, ribosome-associated proteins RaiA and Sra were also heavily synthesized. Some profiles stop at 15 h because H/L ratios could not be determined at later time points (B) Estimates of the amounts of newly synthesized proteins based on iBAQ in the heavy SILAC channel. In contrast to persistence, the amounts of newly synthesized ribosomal proteins are similar to the amounts of newly synthesized RaiA and Sra proteins. The estimated amount of HflX is about 100 times lower.

Almost all detected ribosomal proteins showed synchronized label incorporation during resuscitation (Fig. 4A). Two notable exemptions were the ribosomal proteins L31-type B and L36-2, that showed markedly higher label incorporation compared to other ribosomal proteins. Approximately 10 h after *hipB* induction, the levels of these two proteins surpassed the levels of their paralogs L31 and L36, pointing to a rearrangement of the large ribosome subunit at the entry of the stationary phase (Fig. S2D). This observation, which was recently reported in another study (43), demonstrates the potential of the approach used to detect dynamics of protein complexes in a rapidly changing biological system. Resuscitation was also marked by induction of HflX that correlated with increased synthesis of ribosomal (and other) proteins (Fig. 4B and Data Set S1, sheet 10). Of note, the synthesis of other hibernation and stress factors such as RaiA and Sra did not diminish during resuscitation; however, as opposed to persisting cells, they were not in excess compared to newly synthesized ribosomal proteins.

## DISCUSSION

Numerous studies have shown that bacterial persisters are transcriptionally and metabolically active (9, 13, 44, 45); however, protein synthesis during antibiotic persistence was so far rarely addressed (16, 28). Our study complements previous studies by providing a comprehensive and time-resolved analysis of protein synthesis and turnover during antibiotic persistence and, for the first time, during resuscitation.

In agreement with previous studies (27–29), we found that persister cells generated by ectopic HipA production are actively synthesizing new proteins during long-term ampicillin treatment, albeit at very low levels. During persistence, ribosomal proteins and major proteases involved in general protein turnover were predominantly synthesized, while proteins involved in anabolic pathways and cell division were barely detectable, as expected for a slow-growing or static cell population. RpoS, the sigma factor of the general stress response, was also synthesized at a detectable level, consistent with a condition requiring the production of factors essential in coping with the stress associated with cell stasis. Accordingly, RpoS-dependent factors were also produced, both during persistence and during resuscitation. Remarkably, we observed relatively high synthesis rates of regulatory factors associated with the translation apparatus, both during persistence (RaiA and Sra) and during resuscitation (Sra). During cell stasis and starvation, a large fraction (∼50%) of the ribosomes are known to be degraded, while another fraction is stabilized in an inactive state (46). Inactivation and stabilization of ribosomes during nutritional stress occur by at least two different mechanisms: (i) dimerization of 70S monomer ribosomes to 100S particles mediated by ribosome modulation factor (RMF) plus hibernation-promoting factor (HPF) (47, 48) or (ii) stabilization and inactivation of 70S monomer ribosomes by ribosome-associated inhibitor A (RaiA) (49–51). The findings that RaiA synthesis was significantly stimulated in persister cells and that the *raiA* deletion strain exhibited a lower level of HipA-induced persistence indicated that RaiA plays a role in persistence, probably by stabilizing ribosomes or by reducing translational errors (52) or both. The relatively mild phenotype exhibited by the *raiA* mutant strain during HipA-induced persistence may be due to the redundant mechanisms conferring ribosome stabilization during stasis. This proposal is supported by the observation that RaiA and HPF act synergistically to promote starvation survival in Vibrio cholerae (53). Similarly, the greatly elevated synthesis rate of Sra during persistence and resuscitation led us to test an *sra* deletion strain. In this case, the phenotype was even stronger during HipA-induced persistence, and the *sra* deletion strain also exhibited a reduced level of persistence compared to that of the WT even without the induction of *hipA*. Sra binds tightly to the 30S ribosomal subunit but is present at substochiometric levels relative to the ribosomes (∼1:10) in exponential growth, while this ratio increases in stationary phase (∼4:10) (54). Transcription of *sra* is stimulated in stationary phase by both (p)ppGpp, RpoS, and cyclic AMP (cAMP), factor for inversion stimulation (FIS), and integration host factor (IHF), consistent with the increased level relative to the ribosomes (54, 55). The absence of a clear phenotype of an *sra* deletion strain has previously made it difficult to understand the function of Sra (54, 56). Here, we present the first clear phenotype of a strain lacking Sra, an observation that may facilitate further analyses of the biological function of this protein. At the very onset of resuscitation, cells restored almost the entire translation machinery and restarted their metabolism by inducing glycolysis to convert glucose from the fresh medium into energy used for regrowth. Of note, we recently made a similar observation in resuscitation of chlorotic cyanobacteria (57), pointing to the existence of a common program for awakening of dormant-like cells. Two chaperones (DnaK and GroL) and transcription-related proteins (Rho, DeaD, and Pnp) that are involved in RNA metabolism and degradation were newly synthesized during early response, possibly regulating transcription of specific genes. Because these proteins were also detected to be synthesized during persistence, it is possible that the processes common to persistence are still active during the initial phase of the pulse-labeling. The delay in the wake-up from dormancy was observed previously and depends on several factors, such as the composition of the outgrowth medium or the frequency of persister cells (12, 58).

Molecular investigation of persistence is challenging, and we note that our approach is not free of experimental bias. Although the model that we used—induction of *hipA* and *hipB* genes of E. coli—has been extensively used in previous seminal studies of bacterial persistence (19–24), several uncertainties with this model still remain. First, after *hipA* induction, we used the lytic antibiotic ampicillin to enrich for persisters by physically eliminating sensitive cells; this treatment leads to a heterogenous population of cells that includes viable but nonculturable (VBNC) cells. Although VBNC cells and persister cells are both tolerant to ampicillin and according to some authors present a “dormancy continuum” (59), the exact nature of VBNC cells and their relationship to persisting cells are still debated (60). We note that our present experimental design does not allow us to discriminate between different kinds of persisting cells; however, in future experiments, dynamic SILAC can be combined with methods that can separate VBNC cells from viable, culturable cells and provide further insights into differences that underline these two cell types. Another, more general question is whether our *hipBA* model in combination with ampicillin treatment is representative of all bacterial persisters. As with every model, this is likely not the case; however, our results should be analyzed and interpreted in the context of several previous studies that used the same or similar model to investigate persistence (cited above).

On the technical end, a limitation of our approach is contamination of persister cells with the unlabeled pool of proteins that originate from dead (lysed) cells. Although these proteins can be easily discriminated according to the mass shift of their label, the resulting complexity of the mixture, in combination with a limited dynamic range of mass spectrometric detection, has led to decreased sensitivity of the measurements and lower proteome coverage compared to a usual shotgun proteomics experiment. This issue could be circumvented by an additional enrichment step to isolate persister cells from the batch culture, for example by using a long enzymatic treatment that targets the cell membrane (18) or centrifugation (30). However, such approaches would impose a significant stress to the cells, resulting in proteome changes that could finally lead to data misinterpretation. We cannot exclude the possibility that even gentle filtration of the bacterial culture, performed to induce resuscitation and introduce the Lys8 label, introduced some stress to the cell. The alternative could be persister isolation using flow cytometry based on green fluorescent protein (GFP) expression from plasmid carrying specific promoters of genes associated with dormancy, as reported previously (24, 61). Ideally, the enrichment should not be performed on living cells, but rather on isolated proteins that incorporated the label. For this, approaches based on click chemistry could be used, such as the metabolic labeling with methionine analogue l-azidohomoalanine (AHA) with subsequent attachment of an affinity tag to the reactive azide group and enrichment of the labeled proteins by affinity chromatography (62). However, in our experience, the application of AHA interferes significantly with translation and causes severe growth defects, which is not compatible with our study.

As mentioned above, application of labeled amino acids to study protein synthesis and turnover is often hampered by a limited dynamic range of detection in a mass spectrometer. This means that a very low level of label incorporation will not be detected in a high background of mass spectrometry (MS) signals originating from unlabeled peptides, making detection of label incorporation more likely in abundant proteins. Although the reasons for this are not only technical, as proteins involved in translation and essential proteins are usually abundant, this could be ameliorated by the addition of stable isotope-labeled peptide standards or even the whole-cell lysates as reported in previous analyses of low abundant posttranslational modifications (63). A further complication concerns the recycling of amino acids released from degradation of pre-existing proteins into the medium, which causes a dilution of the labeled amino acid pool with unlabeled amino acids and results in apparently lower turnover rates that cannot truly describe actual values. These values can be corrected using a recycling factor that can be calculated by measuring label incorporation in partially labeled missed cleaved peptides (25). Although these issues may influence the overall sensitivity and accurate estimation of individual protein turnover rates, we do not expect that they lead to a false detection of the newly synthesized proteins.

## MATERIALS AND METHODS

### Bacterial strains and plasmids.

E. coli strains and plasmids used in this study are listed in Table 1. Due to a high toxicity of HipA protein, the *hipA* gene was cloned into the expression plasmids together with a Shine-Dalgarno sequence in which the spacer between the Shine-Dalgarno sequence and the start codon was changed to decrease the translation efficiency of HipA (67). In Table 1, sd8 specifies a consensus sequence AAGGAA with a spacer of eight nucleotides to the GTG start codon. Oligonucleotides used are listed in Table 2.

**TABLE 1:** List of bacterial strains and plasmids

Strain or plasmid	Genotype	Reference
Strains		
MG1655	Wild-type E. coli K-12	64
Δ*lysA*	MG1655 Δ*lysA*::*FRT* P1 transduction from BW25113 *lysA*::*FRT*::*aphA*::*FRT* (Keio collection) into MG1655 and removed by flippase	This work
* sra*::Kan^r^	MG1655 *sra*::*FRT*::*aphA*::*FRT* P1 transduction from BW25113 *sra*::*FRT*::*aphA*::*FRT* (Keio collection) into MG1655	This work
* raiA*::Kan^r^	MG1655 *raiA*::*FRT*::*aphA*::*FRT* P1 transduction from BW25113 *raiA*::*FRT*::*aphA*::*FRT* (Keio collection) into MG1655	This work
Plasmids		
pBAD33	p15-derived expression plasmid, *cat*, *araC*, P_BAD_ promoter, Cm^r^	65
pNDM220	R1-derived expression plasmid, p_A1/O4/O3_ (p*_lac_*), *lacI^q^*, *aphA* (Km^r^)	69
pGOOD	pTrcHisB- and pACYC184-derived plasmid, *lacI*, TetR	66
pBAD33::*hipA* (pEG5)	pBAD33 P_BAD_::sd8 *gtg hipA*	19
pEG220::*hipB*	pEG220 P*_lac_*:: sdopt::*hipB*, Km^r^	This work
pGOOD::p*sra*	pGOOD, *sra* and its native promoter cloned under *lacI* promoter upon digestion with BglII	This work

**TABLE 2:** List of DNA oligonucleotides

Primer	Sequence (5′ to 3′)
EG287	TTAAAAATGAAGTTTTAAATCAATCTAAAGTATATATGAGTAAACTTGGTCTGACAGTTATATGGACAGCAAGCGAACCG
EG288	AGGTGGCACTTTTCGGGGAAATGTGCGCGGAACCCCTATTTGTTTATTTTTCTAAATACATCAGAAGAACTCGTCAAGAAG
EM67	GACTGGGCACAACAGACAAT
EM68	GGATGATCTGGACGAAGAGC
EG239	CATAGACTCGACATAAATCG
EM172	CCCCCGGATCCGTCGACTCAAGGAGTTTTATAAATGATGAGCTTTCAGAAGATCTA
EM173	CCCCCGAATTCTTACCACTCCAGATTTTGCTGTT
raiA.del.KO-f	AGACGGGAAGACAAGAGGT
raiA.del.KO-r	GCGTTGGCGATACACTCA
sra.del.KO-f	CGCAGGCAATGGTGTTTAA
sra.del.KO-r	CTACGGCGATGTTGTCCT
psra.pGOOD-for	CGAAGGCGAAGCGGCATGCAGGTGCTATGCTTGATCGGCA
psra.pGOOD-rev	CCCATATGGTACCAGCTGCATTACTTTTCAGCGGGGCGTT
pGOOD.seq-for	GCCGCCAGGCAAATTCTGT
pGOOD.seq2-rev	CATCACCGAAACGCGCGA

The *lysA*::*FRT*::*Kan*::*FRT* deletion strain from the Keio collection (68) was P1 transduced into E. coli MG1655. The resulting mutation was verified by PCR diagnostics using primers EG239 and EG240 and then transformed with pCP20 to induce the flippase which removes the Kan^r^ resistance cassette by recombination, resulting in MG1655 *lysA*::*FRT* mutant. The *sra*::*FRT*::*Kan*::*FRT* and *sra*::*FRT*::*Kan*::*FRT* deletion strains from the Keio collection (68) were P1 transduced into strain MG1655.

Plasmid pEG220 was constructed to change the kanamycin cassette of the expression plasmid pNDM220 (69) to ampicillin. The ampicillin cassette was amplified with primers EG287 and EG288 from pKD4. Then, DY331 strain has been electroporated with PCR fragment following the procedure from D. Court’s laboratory (https://redrecombineering.ncifcrf.gov/protocols/). The correct insertion of the ampicillin cassette has been verified using primer EM67 and EM68 matching in the insertion in the pNDM220. Then, the kanamycin cassette and the surrounding region of the newly constructed pEG220 were sequenced using primers EM67 and EM68.

For construction of plasmid pEG220::*hipB*, the *hipB* coding region with an optimized Shine-Dalgarno sequence was PCR amplified using primers EM172 and EM173. The resulting DNA fragment was digested with BamHI and EcoRI and ligated in pEG220 digested with the same enzymes.

For construction of plasmid pGOOD::*psra*, the plasmid was digested with BglI and Mph1103I, and the fragment containing the original *lac* promoter was removed. The fragment containing the *sra* gene and its native promoter was amplified with psra.pGOOD-for and -rev and inserted into the same sites. The plasmid was sequenced with pGOOD.seq-for and pGOODseq2-rev.

### Persistence analysis.

A single colony was inoculated into liquid media (Luria broth or M9 minimal medium) supplemented with 0.4% glucose and 25 μg/ml chloramphenicol for maintenance of pBAD::*hipA* and grown overnight. When necessary, 50 μg/ml kanamycin was added for selection of *sra*::*kan* and *raiA*::*kan*. Overnight cultures were diluted to a starting OD_600_ (optical density at 600 nm) of 0.08; at an OD_600_ of 0.3, HipA was induced with 0.2% (wt/vol) l-(+)-arabinose for 1 h, followed by 100 μg/ml ampicillin (Fig. 2). For analysis in M9 medium, bacteria were grown to an OD_600_ of 0.4 and induced for 3 h with 0.2% (wt/vol) l-(+)-arabinose, followed by 100 μg/ml ampicillin (see Fig. S1 in the supplemental material). To analyze persisters under native HipA expression levels (Fig. 2), overnight cultures were diluted in LB at an OD_600_ of 0.08, followed by the addition of 100 μg/ml ampicillin at an OD_600_ of 0.5. Cells were harvested at the indicated time points (Fig. 1), pellets were washed with phosphate-buffered saline, and serial dilutions were plated on LB agar plates supplemented with 0.4% (wt/vol) glucose. Experiments were performed in triplicate. Data were visualized using Prism 8 (GraphPad).

### P1 phage preparation and transduction.

A single colony of donor strain was inoculated in LB broth supplemented with 50 μg/ml of kanamycin and grown overnight. For lysate preparation, the overnight donor culture was diluted 1:100 into 5 ml of 0.2% (wt/vol) glucose and 5 mM CaCl_2_ and incubated 30 to 45 min at 37°C with shaking. One hundred microliters of P1 phage stock was added to the growing culture and incubated, with shaking, until the culture lysed (∼3 h). To complete cell lysis, about 150 μl of chloroform was added to the shaking culture. The culture was pelleted via centrifugation for 10 min at ∼9,200 × *g* at 4°C, and the supernatant containing the phages was filtered over a 0.45-μm sterile filter. For P1 transduction, the E. coli recipient strain was inoculated in 5 ml LB broth overnight at 37°C. The following day, 1.5 ml of the cell culture was pelleted by centrifugation for 2 min at maximum speed at room temperature, and the pellet was resuspended in P1 salt solution (10 mM CaCl_2_, 5 mM MgSO_4_), at half of the original volume. One hundred microliters of cells was mixed with different amounts of the desired P1 lysate (1, 10, and 100 μl), and the phage was allowed to adsorb for 30 min at room temperature. One milliliter of LB broth plus 200 μl of 1 M sodium citrate was added to the cell/phage mix and incubated for 1 h at 37°C. Cultures were then centrifuged, supernatants were removed, and the pellets were resuspended in LB medium. Resuspended cells were plated on LB agar plates supplemented with 50 μg/ml kanamycin. Single colonies were streaked on fresh selective plates and confirmed by PCR.

### Cell growth and SILAC labeling.

Cells were grown in 100 ml of M9 minimal medium (50 mM Na_2_HPO_4_, 22 mM KH_2_PO_4_, 8.6 mM NaCl, 18.7 mM NH_4_Cl, 1 mM MgSO_4_, 0.1 mM CaCal_2_, 0.0001% [wt/vol] thiamine) supplemented with 0.4% (vol/vol) glycerol, 25 μg/ml chloramphenicol for maintenance of the pBAD33::*hipA* plasmid, and 50 μg/ml kanamycin for maintenance of pEG220::*hipB* plasmid. Cultures were grown in batch at 37°C shaking at 200 rpm. Overnight cultures were prepared from a single colony and grown for 20 h in M9 medium supplemented with 0.4% (wt/vol) d-(+)-glucose to repress the P_BAD_ promoter. Overnight cultures were diluted to an OD_600_ of 0.01 to 0.02 in fresh M9 medium without glucose and antibiotics. For HipA-induced persistence, wild-type E. coli K-12 MG1655 strain was used. The expression of *hipA* was induced with 0.2% (wt/vol) l-(+)-arabinose at an OD_600_ of ≈0.4. After 3 h of *hipA* expression, cultures were treated with 100 μg/ml ampicillin for 19 to 22 h to kill nonpersister cells and 0.4% glucose to repress transcription of the P_BAD_::*hipA* promoter fusion. A pulse of 0.025% (wt/vol) stable isotope-labeled lysine derivative ^13^C_6_^15^N_2_ l-lysine (“heavy” lysine, Lys8, and K8; Cambridge Isotope Laboratories) was then added in the presence of ampicillin, and 2-ml portions of cultures were harvested in intermittent time intervals (10 min to 24 h) by centrifugation at 4°C. A time point before the pulse of Lys8 (0 h) was harvested as a control. For the resuscitation experiment, an MG1655-derived strain lacking *lysA* (Δ*lysA*) was used. Growth media were supplemented with 0.025% (wt/vol) natural l-lysine (“light” lysine, Lys0, and K0; Sigma-Aldrich). The expression of *hipA* was induced with 0.2% (wt/vol) arabinose at an OD_600_ of ≈0.4. After 3 h of *hipA* expression, cultures were treated with 0.4% glucose and 100 μg/ml ampicillin for around 22 h. To induce resuscitation, cultures were first quickly filtered using prewarmed 1-liter Corning filter system (0.22-μm pore size) to remove ampicillin, Lys0, and dead cells. Immediately after, cells were quickly recovered in prewarmed medium supplemented with 25 μg/ml chloramphenicol, 50 μg/ml kanamycin, 0.4% glucose, 0.025% Lys8, and 2 mM β-d-1-thiogalactopyranoside (IPTG) for the induction of the P*_lac_*::*hipB* fusion. Cells were harvested at intermittent time intervals (10 min to 30 h) by centrifugation at 4°C. A time point before filtering and Lys8 pulse (0 h) was harvested as a control.

### Cell lysis and sample preparation.

Collected cell pellets were lysed in a lysis buffer (40 mg/ml sodium dodecyl sulfate [SDS], 100 mM Tris-HCl [pH 8.6], 10 mM EDTA, and Complete protease inhibitors [Roche; 1 tablet per 10 ml]) and sonicated three times for 30 s at 40% amplitude. The cellular debris was pelleted by centrifugation at 15,000 × *g* for 30 min at room temperature, and proteins were precipitated from the supernatant with chloroform and methanol. Protein pellets were dissolved in a denaturation buffer (6 M urea, 2 M thiourea, and 10 mM Tris [pH 8.0]). Protein concentration was determined using standard Bradford assay (Bio-Rad). A 10-μg protein aliquot from each time point was reduced using 1 mM dithiothreitol for 1 h and alkylated with 5.5 mM iodoacetamide for an additional hour in the dark. Proteins were digested with endoproteinase Lys-C (1:100 [wt/wt]) for 3 h, diluted with 4 volumes of 20 mM ammonium bicarbonate (pH 8.0), and digested overnight with Lys-C (1:100 [wt/wt]) at room temperature. The reaction was stopped by lowering pH to 2 with trifluoroacetic acid. Peptides were purified via stage tips (70) as described previously (20). Briefly, before each liquid chromatography coupled to tandem mass spectrometry (LC-MS/MS) measurement, samples were desalted and purified on reversed-phase C18 stage tips (Empore), in-house prepared, previously activated with methanol, and equilibrated with solvent A* (2% [vol/vol] acetonitrile, 1% [vol/vol] formic acid in water). Up to 10 μg of peptides was loaded onto the membrane, washed with solvent A (0.1% [vol/vol] formic acid in water), and then eluted with 50 μl of solvent B (80% [vol/vol] acetonitrile and 0.1% [vol/vol] formic acid in water) and concentrated by vacuum centrifugation. The sample volume was adjusted with solvent A and final 10% (vol/vol) of solvent A*.

### LC-MS/MS measurement.

Samples from each time point were separated by the EASY-nLC 1200 system (Thermo Scientific). Peptide separation was performed by reversed-phase chromatography on the in-house packed analytical column (20 cm × 75 μm, 1.9 μm ReproSil-Pur C18-AQ particles [Dr. Maisch]). Peptides were loaded onto the column at a flow rate of 700 nl/min of solvent A (0.1% [vol/vol] formic acid), and a constant temperature of 40°C under maximum back-pressure of 850 bars. Peptides were eluted using 116-min segmented gradient of 10 to 50% solvent B at a flow rate of 200 nl/min. Peptides were infused directly from the column tip into the on-line coupled Q Exactive HF mass spectrometer (Thermo Scientific) using a nanoelectrospray ion source (Thermo Scientific) at a voltage of 2.3 kV and a temperature of 275°C. Positively charged peptides were analyzed in a data-dependent acquisition mode, in which one full scan and subsequent MS/MS scans of 12 (Top12 method) most abundant precursors (isolation window of 1.4 *m/z*) were recorded in a mass range from 300 to 1,650 *m/z*. To prevent repeated fragmentation, the masses of sequenced precursors were dynamically excluded for 30 s. Only precursors with assigned charge states of ≥2 and ≤5 were considered for fragmentation selection. Full scans were acquired with a resolution of 60,000 at *m/z* 200, the maximum injection time of 25 ms, and the automatic gain control (AGC) target value of 3 × 10^6^ charges. The higher energy collisional dissociation (HCD) fragmentation was achieved at normalized collision energy of 27% and intensity threshold of 1 × 10^5^. MS/MS spectra were acquired with a resolution of 30,000, the maximum injection time of 45 ms, and the AGC target value of 1 × 10^5^ charges.

### MS data processing and analysis.

Acquired raw data were processed with MaxQuant software (version 1.5.2.8) (71) using the default settings if not stated otherwise. Raw files of particular experiments were processed separately. The built-in Andromeda search engines searched MS/MS spectra against fragment masses of peptides derived from an E. coli K-12 reference proteome (taxonomy identifier [ID] 83333) containing 4,324 entries (UniProt, release 2017/12) and a list of 245 common contaminants. The minimum required peptide length was set at 7 amino acids with the maximum of two missed cleavage sites allowed for endoproteinase Lys-C. For a protein to be quantified, two occurrences of the protein H/L ratio were required per time point (the multiplicity was set at two with Lys8 specified as the heavy label). Carbamidomethylation of cysteine was set as a fixed modification, and protein N-terminal acetylation and methionine oxidation as variable modifications. The precursor ion mass tolerance was set at 20 ppm in the first search and at 4.5 ppm in the main search. Peptide and protein identifications were filtered using a target-decoy approach with an estimated false discovery rate (FDR) of less than 1.3% at the protein level and less than 0.4% at the peptide level (72). Proteins identified by the same set of peptides were combined into a single protein group. For protein quantification, at least two peptide ratio counts were required.

Data analysis was performed using Microsoft Excel and Perseus software (version 1.5.6.0) (73). Nonnormalized protein H/L ratios from the MaxQuant proteingroup.txt result file were used for protein quantification. All contaminants, reversed hits, and proteins identified only by modification were removed. To simplify the data, the data sets from three biological replicate measurements were combined as a union, in which protein H/L ratios in each of the time points were kept if measured in only one replicate (class I), a mean was calculated if measured in two replicates (class II) or in three replicates (class III). To reduce the number of data points and missing values, time points were pooled into time bins. For that, the median protein H/L ratio of respective time points was calculated and assigned to a specific time bin. Proteins were then ranked based on their H/L ratio within each time bin and displayed in a heatmap-like representation sorted by the maximal H/L ratio across time bins. Gene annotation and KEGG enrichment analysis were performed using the DAVID tool (version 6.8) with default parameters and the background of all quantified proteins in particular experiments (74).

### Temporal analysis of label incorporation.

MaxQuant processed data were imported in the R environment (75) to perform a time series analysis. The heavy label incorporation was calculated by dividing the heavy label intensity by the total (heavy plus light) intensity for each protein. Reverse hits, potential contaminants, and proteins identified only by sites were filtered out. The median of three replicates was computed per time points. Proteins with missing values were discarded from further analysis. The Euclidean distance between proteins was computed and used to generate the hierarchical clustering via Ward’s minimum distance using the stats R package. Time period resolution was achieved with minimal overlap when using eight cluster groups (*k* = 8). The resulting heatmap was drawn using the gplots R package (76), while cluster protein profiles over time were generated using the ggplot2 R package (77). For each cluster, the time needed to reach 50% heavy label incorporation was inferred by intersecting the 50% incorporation with the smoothed conditional mean. The list of proteins belonging to each cluster were imported into Perseus software suite (version 1.6.5.0) (73). The proteins were functionally annotated with Gene Ontology, KEGG, Pfam, GSEA, UniProt keywords, Corum, Interpro, Reactome, and EC functional resources. Each cluster was tested for function category over- or underrepresentation using a Fisher exact test (FDR ≤ 0.1).

### Reactive web application for data browsing.

To allow the scientific community to browse our data sets, we developed a Shiny application coded entirely in the R programming language (78). Our application, called PCTsee, allows user to load different data sets, which are available through a drop-down widget. It is then possible to select gene name or protein ID of interest in order to display their abundance value. The abundance values have been computed by the MaxQuant software during LC-MS/MS data processing (e.g., intensity, ratio). The user selection will result in a protein abundance profile plot, as well as a table containing some information about the selected protein (e.g., protein names, sequence coverage). Users can interact further with the displayed plot to zoom, rescale, or remove samples due to the plotly R package (79). The PCTsee application is available at the following address: https://pctsee.pct.uni-tuebingen.de.

### GO_term enrichment.

Gene ontology analysis of the proteins actively synthesized during resuscitation was performed with the online software DAVID (https://david.ncifcrf.gov/tools.jsp). Enrichment was performed comparing the gene names quantified proteins against the ones of identified proteins. DAVID was used with its preset with similarity overlap of 3 and threshold of 0.5, initial and final group membership of 3 and multiple linkage of 0.5, and enrichment thresohold at EASE 1.

### Calculation of protein turnover rates.

Protein turnover rate (*k*) was calculated by linear regression of natural logarithm of protein SILAC H/L ratio over time as described previously (25) using the following equation independent of the growth rate:

$$k\;=\;\frac{\sum_{i=1}^{m}{\text{log}}_{e}(r_{t_{i}}+1)t_{i}}{\sum_{i=1}^{m}t_{i}^{2}}$$

where *m* is the number of time points ($t_{i}$) and $r_{t_{i}}$ is the protein H/L ratio measured in a time point $t_{i}$. The half-life of a protein ($T_{1/2}$) was calculated by dividing the natural logarithm with the turnover rate *k*:

$$T_{1/2}\;=\;\frac{{\text{log}}_{e}2}{k}$$

Protein H/L ratios derived from a union of three replicates were used for the calculation and only proteins with H/L ratio measured in >4 time points were considered. As a quality check of linear regression, the coefficient of determination (*R*^2^) was required to be >0.70 to ensure good curve fitting and reliable turnover rate estimation. All proteins with determined protein turnover rates are reported in Data Set S1, sheet 1, in the supplemental material.

### Data availability.

The mass spectrometry proteomics data have been deposited to the ProteomeXchange Consortium via the PRIDE (80) partner repository with the data set identifier PXD018153. All other data needed to evaluate the conclusions in this paper are present in the paper or the supplemental material.
